# Endothelial glycocalyx in acute care surgery – what anaesthesiologists need to know for clinical practice

**DOI:** 10.1186/s12871-019-0896-2

**Published:** 2019-12-20

**Authors:** David Astapenko, Jan Benes, Jiri Pouska, Christian Lehmann, Sufia Islam, Vladimir Cerny

**Affiliations:** 1 Department of Anaesthesiology and Intensive Care Medicine, University Hospital Hradec Kralove, Hradec Kralove, Czech Republic; 2 Faculty of Medicine in Hradec Kralove, Charles University, Prague, Czech Republic; 3 Centrum for Research and Development, University Hospital Hradec Kralove, Hradec Kralove, Czech Republic; 4 Department of Anaesthesiology and Intensive Care Medicine, University Hospital Plzen, Pilsen, Czech Republic; 5 Faculty of Medicine in Plzen, Charles University, Prague, Czech Republic; 6 Biomedical centrum, Faculty of Medicine in Plzen, Charles University, Prague, Czech Republic; 7 Department of Anaesthesia, Pain Management and Perioperative Medicine, Dalhousie University, Halifax, NS Canada; 8 Department of Microbiology and Immunology, Dalhousie University, Halifax, NS Canada; 9 Department of Pharmacology, Dalhousie University, Halifax, NS Canada; 10 Department of Physiology and Biophysics, Dalhousie University, Halifax, NS Canada; 11 Department of Computer Science, Dalhousie University, Halifax, NS Canada; 12 Department of Pharmacy, East West University, A/2 Jahurul Islam Avenue, Dhaka, Bangladesh; 13 Departments of Anaesthesiology, Perioperative and Intensive care medicine, J.E. Purkinje 21 University, Masaryk Hospital Usti nad Labem, Socialni pece 3316/12A, 400 11, Usti nad Labem, Czech Republic

**Keywords:** Endothelial glycocalyx, Acute care surgery, Fluid therapy, Transfusion, Major trauma, Anaesthesia

## Abstract

The endothelial glycocalyx (EG) is the thin sugar-based lining on the apical surface of endothelial cells. It has been linked to the physiological functioning of the microcirculation and has been found to be damaged in critical illness and after acute care surgery. This review aims to describe the role of EG in severely injured patients undergoing surgery, discuss specific situations (e.G. *major* trauma, hemorrhagic shock, trauma induced coagulopathy) as well as specific interventions commonly applied in these patients (e.g. fluid therapy, transfusion) and specific drugs related to perioperative medicine with regard to their impact on EG.

EG in acute care surgery is exposed to damage due to tissue trauma, inflammation, oxidative stress and inadequate fluid therapy. Even though some interventions (transfusion of plasma, human serum albumin, hydrocortisone, sevoflurane) are described as potentially EG protective there is still no specific treatment for EG protection and recovery in clinical medicine.

The most important principle to be adopted in routine clinical practice at present is to acknowledge the fragile structure of the EG and avoid further damage which is potentially related to worsened clinical outcome.

## Background

This review aims to describe changes of the EG in critically ill patients requiring acute care surgery to facilitate clinical appreciation and translation of current evidence into clinical practice. The impact of major trauma, acute surgery and selected interventions commonly linked to perioperative care (e.g. fluid therapy, transfusion and specific drugs) on EG integrity will be evaluated. Finally, this review discusses key principles to be adopted by clinicians in order to mitigate EG injury and/or to enhance EG recovery.

### Biochemistry

EG is a carbohydrate-rich mesh covering the apical surface of endothelial cells. It is composed of sulphated glycoproteins connected with sialic acids (heparan sulphate, dermatan sulphate), core proteoglycans (syndecan family, mainly syndecan-1) and non-sulphated glycosaminoglycans connected directly to the cytoplasmic membrane of the endothelial cells (CD 44) [1, 2].

### Physiology

The EG does not only serve as constitutive mechanistic component of the capillary barrier, it has been linked to several important physiological functions of the microcirculation: mechano-transduction [3], blood coagulation [4], immunity [5], antioxidation [6] and interaction with serum proteins [7] and sodium [8].

### Pathophysiology

The delicate nature of the EG makes it extremely vulnerable to damage especially in critical illness such as septic shock [9], ischemia-reperfusion (IR) syndrome, and major trauma [10]. Understanding the role of EG in these conditions is of paramount importance as further damage to the EG can likely play a role in clinical deterioration of the patient, i.e. capillary leakage and interstitial oedema, thrombosis, loss of immune-surveillance and multiorgan failure [11]. Not surprisingly, critically ill patients require often various surgical interventions that may augment existing EG damage.

### Visualization and assessment

**Fig. 1:**
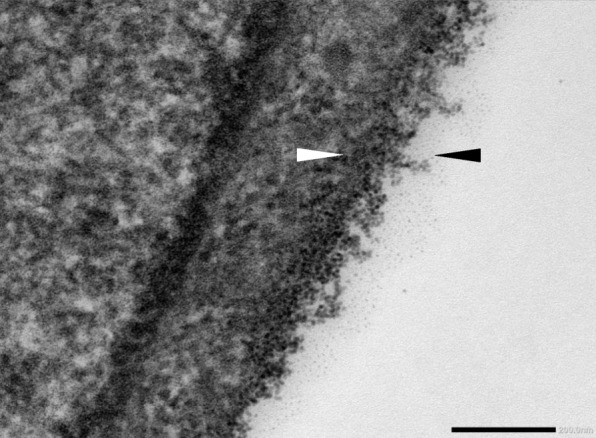
Electron microscopy of endothelial glycocalyx in human umbilical vein endothelial cells by cationized ferritin. Black and white arrows demark the endothelial glycocalyx. The bar represents 200 nm. Image was captured using JEOL JEM-1400Plus transmission electron microscope at the Dept. of Histology and Embryology, Faculty of Medicine in Hradec Kralove, Charles University, Czech Republic. (Courtesy of Dana Cizkova M.D., Ph.D. and Ales Bezrouk Ph.D.)

EG is difficult to visualize and quantitative studies are challenging. First successful electron microscopy of the EG dates back in 1966 [12] although its presence was predicted even earlier [13]. Despite wide usage of transmission electron microscopy (Fig. 1), fluorescence microscopy and intravital microscopy in experimental research [14] these methods are not applicable in clinical patients at the bedside. Clinically, EG can be assessed by Side-stream Dark Field imaging (SDF), or recently Incidental Dark Field imaging (IDF) and specialized software to calculate the so-called Perfused Boundary Region (PBR) which describes the lateral deviation of red blood cells from the central columnar flow and indirectly assesses the extent of EG damage [15]. Second most widely used method to investigate the EG is the biochemical analysis of EG degradation products (e.g., syndecan-1, heparan sulphate, hyaluronan) [16, 17]. A glycocalyx can also be found on other cells, such as red blood cells [18].

### A summary of a search of the existing literature

**Fig. 2:**
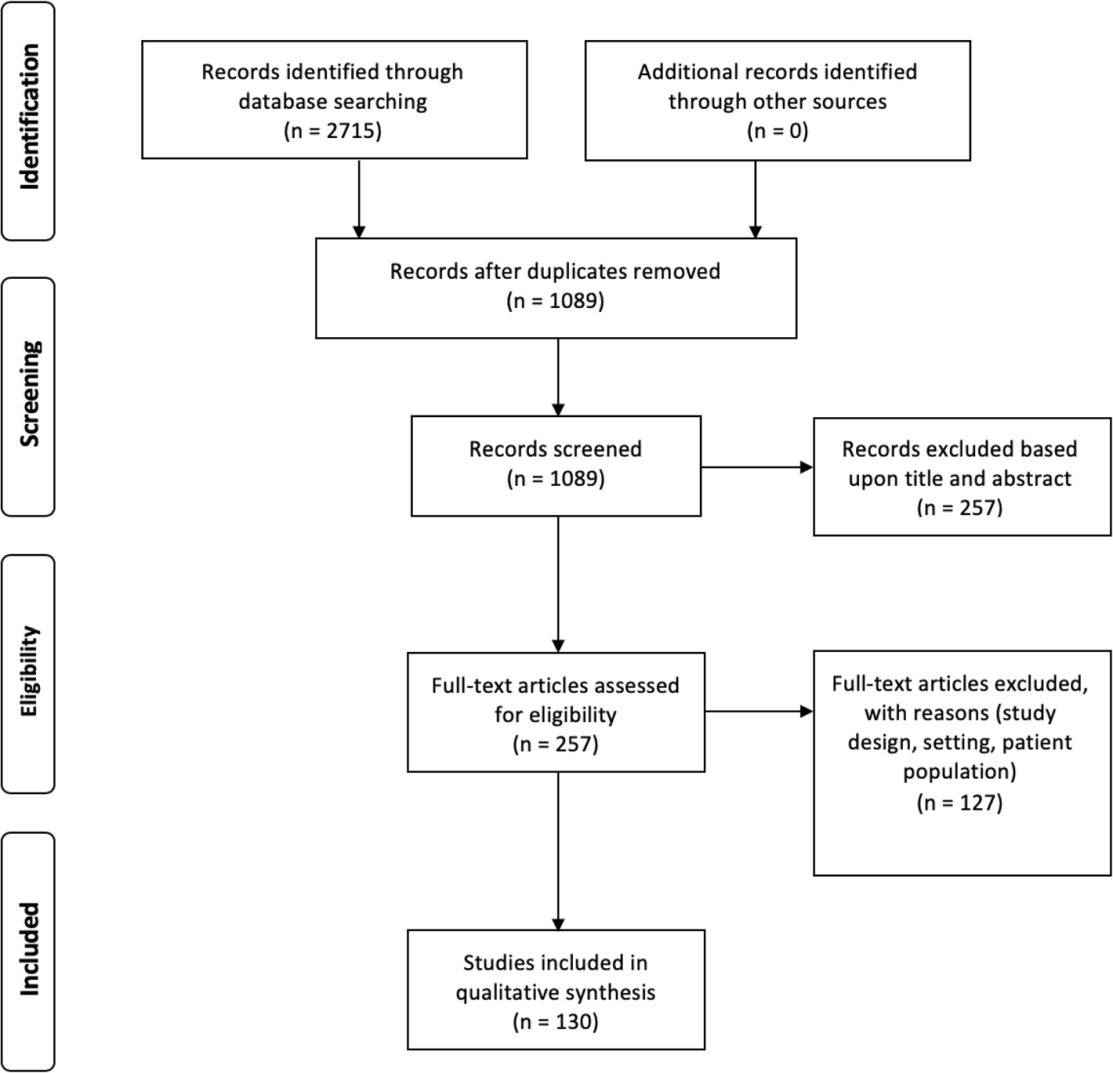
Flow chart of literature search and selection

The PubMed was searched for words: glycocalyx, acute care, trauma, surgery, damage control, anaesthetics, sevoflurane, desflurane, isoflurane, propofol, opioids, fentanyl, morphine, rocuronium, vecuronium, atracurium, pancuronium, catecholamines, phenylephrine, ephedrine, noradrenaline, norepinephrine, adrenaline, epinephrine, insulin, hydrocortisone, antibiotics, cephalosporin, penicillin, quinolones, doxycycline, blood transfusion, transfusion, fresh frozen plasma, plasma transfusion, erythrocytes, blood products, platelets, thrombocytopenia, cryoprecipitate, albumin, coagulation factors, immunoglobulin, sepsis, septic shock. We identified 2715 records. After duplicates removal 1089 papers were screened for relevance and 130 papers were included into the review (Fig. 2). Inclusion criteria were original papers and reviews, English language, topic concerning glycocalyx in clinical and experimental research, publication from 1966 till January 2019.

## Endothelial glycocalyx in acute care surgery

### Endothelial glycocalyx in acute trauma and trauma-related coagulopathy

Major trauma leads to 5.8 millions of deaths worldwide annually [19]. Within the first hours, traumatic brain injury, unsurvivable body disruption and exsanguination are the major causes of death [20] [21]. Despite of extensive research in this field, optimal care of trauma patients remains a challenge. Trauma induces a systemic inflammatory response syndrome (SIRS). SIRS-related stress affects EG integrity by several pathways and mechanisms. Acute hyperglycaemia has been demonstrated by Diebel et al. to take part in trauma-induced EG injury [22]. EG shedding is also promoted by enzymes released from damaged tissue and leukocytes (e.g. matrix metalloproteinase, hyaluronidase, heparanase). Degradation products of EG such as syndecan-1, hyaluronan, and heparan sulphate) have several functions. They activate TLR-2 and TLR-4 receptors as damage associated molecular pattern (DAMP) potentiating the inflammatory response [23] which can even lead to compensatory immunosuppression [24] and higher risk of nosocomial pneumonia in severely injured patients [25, 26]. On the contrary, this microvascular response to trauma is of physiological importance. EG contains nearly 1.5 l of plasma which is ready to replenish intravascular space if needed [27] and thus EG acts as a potent and fast fluid reservoir.

Sensitivity of EG to degradation in this context represents an evolutionary advantage to counteract acute blood loss (in conjunction with activation of sympathetic nervous system keeping vital organs perfused).

The primary insult triggers EG shedding [28] which has been shown to increase with severity of injury. High levels of syndecan-1 were associated with severity of traumatic brain injury (TBI) [29, 30] and increased mortality [16, 31]. Alteration of EG has been also shown in experimental spine injury in rat [32]. In patients with major burns high levels of syndecan-1 were associated with age and fluid requirements [33]. These changes lead to general activation of the endothelium, i.e. traumatic endotheliopathy [34].

**Fig. 3:**
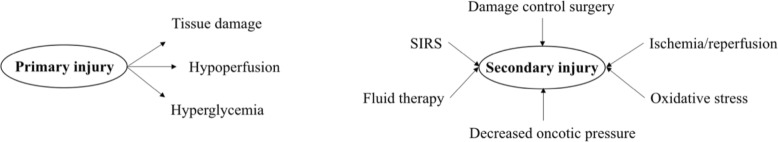
Endothelial glycocalyx is damaged by primary and secondary injury. This figure demonstrates that secondary injury is more diverse and is better influenced

Secondary injury can be induced by SIRS, IR, oxidative stress, and iatrogenic damage due to the inadequate fluid therapy (see below) as well as inadequately performed damage control surgery (Fig. 3). Damage control surgery is meant to treat the “lethal triad” (metabolic acidosis, hypothermia, coagulopathy) rather than correcting anatomy [35] and should be always considered as an intervention aiming to stop ongoing haemorrhage and/or to remove necrotic tissue. One of the techniques used to prevent excessive blood loss is permissive hypotension which has been shown to increase survival and decrease complications [36]. On the other hand, prolonged hypotension leads to impaired microcirculation and EG damage [37] and perioperative lung injury [38].

Blood loss and hemorrhagic shock are closely associated with severe trauma. Optimal fluid management in hemorrhagic shock has been studied extensively in animal models [39, 40] and is discussed later. Filho et al. showed that the EG is damaged also at the venular level of the mesenteric and skeletal muscle microcirculation [41] which might be responsible for further pathophysiologic changes manifesting clinically (especially intestinal failure and spontaneous bacterial peritonitis due to impaired permeability of intestinal wall). Leakage of plasma proteins and subsequent decrease in colloid osmotic pressure further aggravates the EG damage and impaired permeability [28]. Conversely, the degradation of EG seems to be independent of increased permeability in rat model of non-traumatic hemorrhagic shock [42]. Beside transfusion therapy, which is capable of EG modulation (discussed in detail below), valproic acid has been shown to decrease lesion size and volume in rodent model of TBI but increased EG shedding [43].

After major trauma, hypoperfusion and vascular damage cause almost immediately primary endogenous disturbances in the coagulation system known as acute traumatic coagulopathy (ATC) [44]. The cell-based model of hemostasis [45] is the key concept for understanding its pathophysiology as a complex balanced system of pro- and anticoagulant factors (distinct molecules in plasma), various blood cells and finally blood vessels. Fundamentally, there are four separated entities in the pathophysiology of ATC – [1] activated protein C (APC) pathway, [2] endothelial dysfunction (traumatic endotheliopathy), [3] inadequate amount of fibrinogen and [4] platelet dysfunction. Among them, the APC pathway is considered to play an essential role [46]. After tissue trauma, due to increased expression of thrombomodulin on the endothelium and massive thrombin generation (known as “thrombin burst”) thrombin-thrombomodulin complexes arise in large numbers [47]. These complexes dramatically accelerate activation of protein C [48] which in turn has pivotal role in tipping the balance of haemostasis in favour of hypocoagulation. Through inactivating factor Va and VIIa, the APC leads to reduced clot formation and via antagonism of tissue-type plasminogen activator inhibitor (PAI-1) it amplifies clot breakdown.

Altered tissue perfusion represents another characteristic feature of hemorrhagic shock. Naumann et al. [37] demonstrated in 17 trauma victims that endotheliopathy and glycocalyx shedding are the key factors in the altered microcirculatory flow after hemorrhagic shock. Moreover, they measured significantly higher levels of thrombomodulin after trauma versus healthy cohort. EG disruption after trauma was consistently described [16]. Several factors including tissue trauma, inflammation, hypoperfusion and sympathoadrenal activation may result in EG shedding, endothelial activation with expression of anticoagulant proteins on the luminal surface and hyperpermeability. Two potential mechanisms of ATC induced by EG destruction have been identified recently. The first one is a link between EG integrity and APC pathway [31, 49–51] - EG disruption (measured by serum syndecan-1) correlates with increased soluble thrombomodulin level, reduced protein C concentration (indirect marker of elevated APC), elevated vascular endothelial growth factor and degranulation of Weibel-Palade bodies [52] (containing tissue plasminogen activator and angiopoietin 2). Tissue trauma releases tissue plasminogen activator (t-PA) from endothelial cells. Under conditions of increased adrenalin and vasopressin serum levels the t-PA release is augmented [34] leading to hyperfibrinolysis. Furthermore, a connection with other haemostatic systems (immune, sympathoadrenal, etc.) can be presumed, which are linked to coagulation [53] although strong scientific evidence remains to be discovered.

The second possible mechanism of EG-induced ATC is auto-heparinization. EG is made up by glycosaminoglycan macromolecules, out of which heparan sulphate forms the majority. Rehm et al. [54] showed in major vascular surgery patients the connection between disruption of EG and heparan sulphate release. Its heparin-like properties leads to anticoagulation (or endogenous heparinization), which can be detected by TEG or ROTEM [55]. This auto-heparinization appears to be augmented in hemorrhagic shock and can be recognized as a continuum of EG shedding [55–57].

Acute traumatic coagulopathy as a result of endogenous coagulation deficit, can be further worsened by inadequate resuscitation (including hypothermia and haemodilution). It has been also termed as a trauma-induced coagulopathy (TIC), to describe those mechanisms affecting the coagulation following trauma. Thus, trauma care providers should focus on primary endogenous coagulopathy (ATC) as well as support care to avoid secondary TIC. For example, crystalloid overload may lead to transient hypervolemia [58], which can contribute itself to EG disruption and in fact worsen ATC/TIC [59].

Therefore, a rational approach of trauma resuscitation should take not only the substance (specific fluid composition, drugs etc.), but also its amount and other factors (i.e. time, patient’s temperature, serum pH) into account. This approach is crucial, since we do not have specific EG regeneration therapies and the only way to block EG disintegration is early reversal of tissue hypoperfusion and avoiding further progression of shock. Routinely used tranexamic acid might be the sole exception: in vitro protective effect on EG has been demonstrated in oxidative stress [60].

Key clinical targets to prevent further EG damage:

- Effective source control of bleeding, damage control surgery if indicated
- Effective resuscitative measures to restore/maintain adequate tissue oxygenation and perfusion
- Early administration of tranexamic acid
- To avoid worsening precipitating factors of ongoing coagulopathy, especially hypothermia and haemodilution

### Endothelial glycocalyx in acute surgery, anaesthesia and perioperative care

#### Fluid therapy

Patients undergoing acute care surgery are frequently hemodynamically unstable. Therefore, multiple interventions are needed to save their lives. Fluid therapy is still considered the cornerstone of hemodynamic resuscitation [61]; in particular, in patients with hypovolemic/hemorrhagic and septic shock, who represent the vast majority of the high-risk acute care surgery population. Over the years, the number of available resuscitative fluids has decreased [62] because more adverse effects of certain fluids have been discovered [63]. It has been repeatedly demonstrated (both in animal experiments [41, 64, 65] and using laboratory markers of EG disruption in humans) [34, 57, 66] that inflammation, sepsis, trauma, and haemorrhage all lead to EG shedding. The SHINE acronym (shock induced endotheliopathy) has been proposed to describe this pathology common to sepsis, IR and/or traumatic shock states [67].

Based on our current knowledge, SHINE plays an important role in the regulation of endothelial permeability; the so called revised Starling principle [27, 68]. In situations, when the EG is disrupted, the extravascular fluid leak may promote oedema formation with all its consequences. The nature of the disease process and severity of the EG injury may hence play an important role and have implications on the volume needed to regain adequate circulating blood volume. In an observational study in 175 septic shock patients in a single centre emergency department, high levels of syndecan-1 indicated patients with higher risk of intubation (odds ratio of 2.71 (1.33–5.55 95% confidence interval)) after a “large volume” (mean volume of 4 l) fluid resuscitation [69]. The different volume effects of hydroxyethyl starch infusion in blunt and penetrating trauma observed in the FIRST (Fluid In Resuscitation in Severe Trauma) trial may be hypothetically coupled with unequal EG activation though not measured in this study [70]. In another observational trial, serum hyaluronan levels were associated with the cumulative fluid load administered during the emergency treatment of patients with inflammation, sepsis and septic shock [66]. Differences in volume kinetics observed in multiple studies (reviewed in Hahn and Lyons) [71] might all point on our sparse knowledge about the actual effect of fluid therapy and poor understanding of its limits [72].

However, the relationship between EG and fluids is not unilateral. Recently, there has been an increasing number of studies demonstrating that fluid administration itself may lead to EG damage. In normovolemic human volunteers, intravascular expansion using crystalloids [73, 74] increased significantly the hyaluronan serum levels pointing on EG shedding, whereas infusion of 4% albumin and dextran seemed not to have any influence in the latter study [73]. Crystalloid bolus in term parturient also led to increase in EG shedding markers (heparan sulphate and syndecan-1) in another observational study [75]. Atrial natriuretic peptide (ANP) was associated with transient hypervolemia and EG shedding in another human study [58], but did not entirely explain the findings in parturients [75]. Recently, a Slovenian group has demonstrated in patients undergoing elective laparoscopic cholecystectomy that large volume fluid intake (15 ml/kg/hour) led to increase of hyaluronic acid and syndecan-1 levels as compared to restrictive regimen (1 ml/kg/hour) [76]. In all these trials the EG degradation molecules (syndecan-1, hyaluronan or heparan sulphate) were used to study EG shedding. In another study of elective surgical patients our group has demonstrated a transient decrease in EG thickness after crystalloid fluid challenge using intravital real time light reflectance video-microscopy of sublingual microcirculation and PBR calculations [77]. All previous studies were based on human volunteers or elective patients with presumably intact EG and its derangements may be attributed to transient hypervolemia induced by fluid infusion and/or ANP release. Besides, it seems that the concentration of sodium may play important role in EG stability. Martin et al. has recently performed an in vitro study demonstrating EG degradation (both by syndecan-1 serum levels and by fluorescent microscopy) in hypernatremic conditions (160 mEq/L) further worsened by simulated shock conditions [78]. Our group has observed increased PBR thickness in rabbits after infusion of hypertonic 10% saline though not coupled with increased EG-degradation molecule levels possibly explainable by acute volume change in EG layer [79].

In acute care surgery, the situation might be much more complex. The EG is generally damaged by the primary impact and fluids may further aggravate the injury although in some cases restoration is possible. In a second arm of the above-mentioned trial by our group [77] the same crystalloid fluid challenge was performed in resuscitated septic shock patients; the PBR was significantly higher (hence EG thinner) among these patients, moreover the fluid challenge increased the PBR further on. Unlike in the elective surgical population, in septic patients the PBR increase lasted until the end of experiment. In a small animal study of acute pancreatitis, fluid resuscitation to pre-septic baseline vs. fully stroke volume maximalization led to smaller infusion volumes and oedema formation in pancreatic tissue, but also smaller inflammatory activation (interleukin-6) and EG damage (measured by heparan sulphate levels) [80]. In a set of animal experiments with non-traumatic hemorrhagic shock in rats, Torres et al. demonstrated that lactated Ringer, normal saline, and to lesser intense iso-oncotic (5%) albumin solution and hypertonic (3%) saline decrease the thickness of the EG and increase the EG disruption molecules (snydecan-1 and heparan sulphate) [59, 64]. Interestingly volume replacement with allogenic blood products did not have such detrimental impact in both these trials. Similar results were found in a canine model of haemorrhage and shock [65] with the most pronounced EG injury and inflammation activation (measured by IL-6 and IL-8 and IL-10 release) after crystalloid resuscitation as compared to fresh whole blood; artificial colloids (gelatine and hydroxyethyl starch) were somewhat less injurious and almost comparable to whole blood in this trial. It is important to note that the disruptive effect of fluid loading in many of these experiments measured via degradation molecules and vascular permeability did not match entirely [42, 64] pointing to the fact that there may be other hidden factors involved. For instance, spingosine-1-phosphate (a phospholipid normally carried by albumin and produced by red blood cells) has been identified recently as a potential target molecule being able to stabilize the EG matrix [81, 82]. A possible protective effect of iso-oncotic albumin solution has been reported by Jacob et al. in two laboratory studies with isolated heart but didn’t seem to be clinically reproducible [83, 84].

Key clinical targets to prevent further EG damage:

- Avoiding fluid overload
- Avoiding severe hypernatremia
- No direct recommendation regarding the type of solution as well as preference of some molecules (i.e. gelatine, HES, albumin) could be made

#### Blood products

Blood products are classified as blood components (red blood cells, platelets, fresh frozen plasma and cryoprecipitate) or plasma derivatives (albumin, coagulation factors and immunoglobulins). Blood components and selected coagulation factors are often administered during acute surgery due to pre−/intra-operative blood loss and coagulation deficits, namely in the context of the major trauma bleeding [85]. Moreover, endotheliopathy and sympathoadrenal activation may drive hypocoagulability and hyperfibrinolysis in trauma patients [67, 86]. Despite the fact that it is difficult to distinguish EG injury due to critical conditions (e.g. trauma) and due to the effect of a particular blood product, evaluating the effects of blood components on EG integrity is definitely of great interest for clinicians and may broaden our view on the current transfusion practices in various subgroups of patients.

##### Red blood cells transfusion

There are only few clinical studies evaluating the effect of RBC transfusion on various markers of EG integrity as a primary endpoint, most of them evaluate relationship between severity of the illness/injury and various laboratory markers of endothelial damage in different groups of patients. In patients with hematologic diseases, RBC transfusion was associated with reduced EG degradation as assessed by syndecan-1 levels [87], and in severely injured patients soluble vascular endothelial growth factor receptor 1 and syndecan-1 levels correlated with high early and late transfusion requirements [88]. A prospective, observational study revealed, that the combined highest plasma levels of adrenaline, injury severity, shock and in-hospital transfusion were associated with excessively increased syndecan-1 levels [89].

Overall, current evidence supports the possible role of RBC transfusion in modulating EG. However, in the clinical setting of acute patients, effects of other parallel interventions may play a bigger role. Therefore, to our opinion, any scientifically based conclusion for clinical practice cannot be drawn at this stage.

Direct translation to clinical practice except for routine practice and standard measures:

- None

##### Fresh frozen plasma

Current evidence supports the concept of plasma as a key player in protection from endotheliopathy induced by trauma or hemorrhage [90, 91]. The effects of plasma protein administration on glycocalyx thickness of frog mesentery vessels was studied even in early nineties, the total glycocalyx thickness was twice the value seen with Ringer solution [92]. Experimental studies suggest that plasma can repair the endothelial surface by restoring EG and inhibiting shedding of syndecan-1 [90, 91, 93, 94]. A clinical trial evaluating patients undergoing emergency surgery for thoracic aorta dissection found that solvent/detergent-treated pooled plasma reduced glycocalyx and endothelial injury compared to standard fresh frozen plasma (FFP) [94]. A recently published review summarizes extensively the current evidence on the role of plasma in protecting endothelium [95]. Syndecan-1 seems to be a key mediator of possible beneficial effect of plasma on EG integrity, where plasma enhances endothelial syndecan-1 expression in dose dependent manner [96]. While there is extensive preclinical evidence for the ability of FFP in preserving the EG, suggesting a role beyond its current indication as a source of coagulation factors, this evidence is currently lacking for preparations of factor concentrates that are currently marketed and recommended as alternatives. There is currently insufficient clinical evidence upon which to recommend FFP over factor concentrates in this respect, but arguably there is both rationale and equipoise for a randomised controlled trial.

Direct translation to clinical practice except for routine practice and standard measures:

- None

##### Cryoprecipitate

Searching for relevant studies evaluating cryoprecipitate administration in relation to EG retrieved no results.

##### Coagulation factor concentrates

We found one experimental study evaluating the impact of coagulation factor concentrates (CFC) on markers of endothelial cell damage in experimental hemorrhagic shock. Rats were resuscitated with FFP, human albumin, and Ringer’s lactate, supplemented with fibrinogen concentrate or prothrombin complex concentrate. There was no benefit of CFC co-administration on markers of EG shedding. Resuscitation with FFP restored heparan sulphate back to baseline levels [97]. Wu and co-workers recently hypothesize the important role of fibrinogen in stabilizing syndecan-1 on the cell surface and propose interesting pathway for protecting effect of fibrinogen of endothelium [98]. If such barrier effect of fibrinogen on EG confirmed and extrapolated in clinical practice, we would have the other reason to support the early use of fibrinogen in patients with hemorrhagic shock and related endotheliopathy then.

Direct translation to clinical practice except for routine practice and standard measures:

- None

##### Platelets

Platelet adhesion to endothelial cells is important in triggering thrombosis and inflammation. Intact EG seems to be a prerequisite to prevent such adhesion. Our search revealed no studies evaluating platelet transfusion with relation to EG. The role of interaction between platelets transfusion and EG needs to be explored urgently, current knowledge supports the key role of platelets in inflammation and sepsis [99, 100].

Direct translation to clinical practice except for routine practice and standard measures:

- None

Current evidence does not allow any clinically relevant conclusions or recommendations with respect to common transfusion practices. It is clear that there is biological interaction between the endothelium and blood products, as soon as they reach the intravascular compartment during their administration. Nevertheless, such interaction, especially in the setting of acute care surgery, will be affected by several other internal (e.g. baseline EG status) and external factors (e.g. fluid balance, sodium levels) which makes it difficult to predict the effects of particular blood products on EG integrity. On the other side, the concept of plasma administration as an intervention to attenuate endotheliopathy related to trauma (or surgery) seems to be promising and deserves further clinical testing.

#### Specific drugs

Apart from fluid resuscitation and blood products, the most administered drugs in the perioperative setting are anaesthetics, catecholamines, insulin, steroids and antibiotics.

##### Anaesthetics

There are only a few publications on EG effects of anaesthetics. First studies on the acute impact of (local) anaesthetics on EG integrity were published almost 40 years ago. However, those early studies focused on the erythrocyte EG [101, 102]. Aesthetic effects on endothelial EG were only studied in the last decade. The first study on the effects of volatile anaesthetics on EG structure was published by Annecke et al. in 2010 [103]. The authors observed in isolated guinea pig heart preparations, that sevoflurane protects the endothelial EG from IR-induced degradation. In another study in anesthetized pigs, the same authors found, that sevoflurane proves to be superior to propofol in protecting the endothelium from IR injury [104]. Casanova et al. confirmed the findings in the pulmonary circulation [105]. For desflurane or isoflurane, such studies are not available. Unfortunately, the only clinical study in patients so far was not able to reproduce the better protective effects of sevoflurane on endothelial EG compared to propofol during lung surgery (Kim, 2018) [106]. With regard to propofol, Lin et al. reported that high doses of propofol cause an ATP-dependent reduction of EG expression and consequently lead to vascular hyperpermeability due to the loss of endothelial barrier functions [107]. Opioids and muscle relaxants are not studied yet regarding their potential impact on EG. According to the results of our own studies, regional anaesthesia seems to have less impact on EG compared to general anaesthesia, however, such preliminary results must be robustly confirmed by adequately powered clinical trials before any recommendation for particular anaesthesia technique to modulate EG can be made [108].

Direct translation to clinical practice except for routine practice and standard measures:

- None

##### Catecholamines

In acute care surgery, catecholamine administration is often required as a consequence of anaesthetics-induced vasodilation and/or relative or absolute hypovolemia, respectively [109]. The impact of fluid resuscitation and blood product administration on EG was described above. Catecholamines are clinically used to bridge critical situations and stabilize the hemodynamics of the patients. Therefore, they are beneficial to reduce detrimental effects of hypotension on EG integrity. Catecholamines also help to reduce potential negative side effects of fluid therapy such as hypervolemia, which is also known to cause shedding of the EG [110]. Interestingly, in a recent study, Byrne et al. observed a paradoxical increase in vasopressor requirement during fluid resuscitation in experimental septic shock compared to vasopressor only treatment [111]. Combination of fluid therapy with vasopressors did not result in improvements in any of the microcirculatory or organ-specific markers measured in this model. The increase in vasopressor requirement may have been due to EG damage secondary to ANP-mediated EG shedding. Apart from the hemodynamic impact, some investigators studied other direct or indirect effects of catecholamines on the EG. In vitro, Martin et al. treated human umbilical vein endothelial cells (HUVEC) with varying concentrations of norepinephrine or epinephrine [112]. Norepinephrine was associated with significantly greater EG damage and endothelial activation vs. epinephrine treatment groups.

Direct translation to clinical practice except for routine practice and standard measures:

- None

##### Insulin

Hyperglycaemia is a physiological stress response. However, both acute and chronic hyperglycaemia can cause EG damage [2]. E.g., Zuurbier et al. showed in mice with acute hyperglycaemia (25 mmol/l) a sustained increase in EG permeability [113]. In humans, Nieuwdorp et al. reported almost 50% loss of EG volume at a blood glucose level of 15 mmol/l. [114] The same dramatic changes in EG volume can be observed in patients with type I diabetes and chronic hyperglycaemia – approximately a half of the EG volume is lost [115]. The underlying mechanism connecting hyperglycaemia and glycocalyx disruption is not fully understood yet. In a recent review article, Lemkes et al. postulated that hyperglycaemia leads to the formation of reactive oxygen species, which can cause direct EG damage [116]. Therefore, glycaemic control represents not only a metabolic requirement, but also a way to protect the EG. Accordingly, O’Hora et al. were able to demonstrate in anesthetized pigs, that insulin was able to improve vascular reactivity. However, in contrast to their working hypothesis, this was a EG-independent insulin effect mediated through increased NO synthesis [117]. At present, no clinical data regarding insulin effects on endothelial EG setting are available in the acute care surgery. Given the immanent risks of perioperative hypoglycaemia, insulin should be carefully administered and the optimal perioperative blood sugar range is considered to be 5 to 10 mmol/l. [2] Interestingly, in patients with pre-existing diabetes, insulin therapy (in contrast to oral antidiabetic therapy) was shown to be related to higher levels of serum syndecan-1, generally considered as a marker of EG shedding, i.e. damage. However, in the presence of insulin, there is an even larger increase in syndecan synthesis compared to in its absence, which is actually beneficial since syndecan-1 can decline leukocyte–endothelial cell interactions, decrease angiogenesis, reduce inflammatory responses and anti-coagulate, which can protect endothelial cells from damage of inflammation, and slower down the development of micro and macroangiopathy [118].

Key clinical target to prevent further EG damage:

- Avoiding severe hyperglycaemia

##### Steroids

Main indications for the administration of steroids in the acute care surgery setting include anti-oedematous (brain surgery, airway complications), immunosuppressive (transplant), and anti-emetic (PONV) therapies. Furthermore, patients with long-standing, high-dose corticosteroid treatment require usually a “stress-dose” of hydrocortisone. Stress was experimentally induced by Chappell et al. by TNF-alpha infusion into guinea pig hearts causing severe EG destruction in the coronary vessels. Pretreatment with hydrocortisone was able to attenuate these changes significantly [119]. Of similar benefit was the administration of hydrocortisone in ischemia and reperfusion, mitigating inflammation, thus protecting against the ‘low-reflow’ phenomenon [120]. Furthermore, hydrocortisone is recommended in the Surviving Sepsis Campaign guidelines in patients with septic shock refractory to fluids and vasopressors [121].

Direct translation to clinical practice except for routine practice and standard measures:

- Consider stress dose of hydrocortisone

##### Antibiotics

Antibiotics are an integral part of acute care surgery – as perioperative prophylaxis or specific therapy for infections [122]. The action of some antibiotics is closely related to the bacterial glycocalyx [123, 124] which composition is similar to EG. Therefore, it is surprising, that almost nothing is known about the impact of antibiotic treatment on the EG: Lipowsky et al. showed that sub-antimicrobial doses of doxycycline attenuated chemoattractant induced EG shedding through matrix metalloprotease (MMP) inhibition [125]; L-658758, a cephalosporin-based beta lactam, was able to reduce EG shedding by inhibition of neutrophil elastase [126]. Last but not least, renal endothelial EG integrity has an impact on the pharmacokinetics of many antibiotics, which can be important in patients with acute or chronic kidney failure [127].

Direct translation to clinical practice except for routine practice and standard measures:

- None

### Future research directions, new concepts

Current experimental and clinical evidence indicates a clinical potential for the modulation of EG integrity by various means [10]. Research on in vitro*/*in vivo models (HUVEC, rats, guinea pig) showed promising results and several protecting agents and interventions to modulate dysfunctional EG have been identified (Table 1), among them, frequently studied candidates for further research are: sphingosine-1-phosphate [82], hyaluronan [17] and sulodexide [128] (combination of medium long chain heparan sulphate and dermatan sulphate). These agents need to be investigated in properly designed and powered clinical trials to validate clinically relevant benefit for the patients with acute care surgery.

**Table 1:** Endothelial glycocalyx protecting agents

Author, reference	Agent	Description
Diebel [60]	Tranexamic acid	Inhibition of endothelial sheddase activation in HUVEC
Barelli [95]	Fresh frozen plasma	Restoration of endothelial barrier function
Nelson [40]	Human serum albumin	Faster plasma volume expansion in a rat model of hemorrhagic shock
Annecke [103]	Sevoflurane	Decreased transudate formation after IR in guinea pig hearts
Alves [81], Zeng [82]	Sphingosine-1-phosphate	Protecting endothelial mitochondrial integrity, inhibition of syndecan-1 shedding
Astapenko [108]	Regional anaesthesia	Decreased raise in PBR in hip replacement surgery
Chappell [119]	Hydrocortisone	Attenuation of coronary vessel damage after IR in guinea pig hearts
Lipowsky [125]	Doxycycline	Inhibition of MMP in rat mesenteric microcirculation
Carden [126]	L-658758	Inhibition of elastase in isolated rat lungs after IR
Lennon [17]	Hyaluronan	Reconstitution of EG
Broekhuizen [128]	Sulodexide	Reconstitution of EG
Schmidt [129]	Heparin	Inhibition of heparanase

*HUVEC* human umbilical vein endothelial cells, *MMP* matrix metalloproteinase, *PBR* perfused boundary region, *IR* ischemia/reperfusion

## Conclusions

During conditions leading to acute care surgery, EG is damaged by the non-modifiable primary insult. However, acutely injured patients often experience secondary injury, mostly caused by ongoing tissue trauma during surgical preparation, related inflammatory reaction, hypovolemia due to blood loss and other causes. EG protecting approaches during the perioperative period must be based on deep knowledge and understanding of the physiology of the vascular compartment. Even though some interventions are already known as potentially EG protective (e.g. transfusion of plasma, human serum albumin, hydrocortisone, sevoflurane) there is still no specific treatment for EG protection and recovery in clinical medicine to be used during acute care surgery and anaesthesia. The general advise for clinicians seems to be very simple, nevertheless, it is solidly physiologically based and reflecting current evidence: In order to protect EG in perioperative setting, avoid all events that could lead to secondary EG injury, i.e. 1) perform damage control surgery to remove potential sources of sepsis; 2) minimizing surgical time; 3) restore and maintain hemodynamic stability; 4) avoid fluid overload.

## Data Availability

Not applicable.

## Abbreviations

ANP: Atrial natriuretic peptide
APC: Activated protein C
ATC: Acute traumatic coagulopathy
ATP: Adenosine triphosphate
CFC: Coagulation factors concentrate
DAMP: Damage associated molecular patterns
DIC: Disseminated intravascular coagulation
EG: Endothelial glycocalyx
FFP: Fresh frozen plasma
HUVEC: Human umbilical vein endothelial cells
IL: Interleukin
IR: Ischemia-reperfusion syndrome
MMP: Matrix metalloproteinase
NO: Nitric oxide
PAI: Plasminogen activator inhibitor
PBR: Perfused boundary region
PONV: Postoperative nausea and vomiting
RBC: Red blood cells
ROTEM: Rotational thromboelastometry
SIRS: Systemic inflammatory response syndrome
TBI: Traumatic brain injury
TEG: Thromboelastography
TIC: Trauma induced coagulopathy
TLR: Toll-like receptor
TNF: Tumor necrosis factor
t-PA: Tissue plasminogen activator
